# Mental health characteristics and their associations with childhood trauma among subgroups of people living with HIV in China

**DOI:** 10.1186/s12888-021-03658-5

**Published:** 2022-01-05

**Authors:** Dongfang Wang, Qijian Deng, Brendan Ross, Min Wang, Zhening Liu, Honghong Wang, Xuan Ouyang

**Affiliations:** 1 Department of Psychiatry, National Clinical Research Center on Mental Disorders, China National Technology Institute on Mental Disorders, the Second Xiangya Hospital of Central South University, Changsha, 410011 China; 2 School of Psychology, Centre for Studies of Psychological Applications, Guangdong Key Laboratory of Mental Health and Cognitive Science, Ministry of Education Key Laboratory of Brain Cognition and Educational Science, South China Normal University, Guangzhou, China; 3 Faculty of Medicine, McGill University, Montreal, QC Canada; 4 Institute for HIV/AIDS, the First Hospital of Changsha, Changsha, China; 5 Xiangya Nursing School, Central South University, Changsha, China

**Keywords:** People living with HIV, Psychotic-like experience, Anxiety, Depression, Childhood trauma

## Abstract

**Background:**

People living with HIV (PLWH) carry a high risk for mental health problems, which has been extensively reported in the literature. However, an understanding of mental health characteristics in different subgroups of PLWH is still limited. In the present study, we conducted a cross-sectional survey to explore mental health characteristics and their associations with childhood trauma in two major subgroups of PLWH in China.

**Methods:**

A total of 533 PLWH (213 prisoners in the prison system, and 320 outpatients) were assessed using the 8-item Positive Subscale of the Community Assessment of Psychic Experiences (CAPE-P8), Generalized Anxiety Disorder scale (GAD-7), Patient Health Questionnaire (PHQ-9), and Childhood Trauma Questionnaire (CTQ).

**Results:**

From the total sample, 22.0% PLWH frequently experienced psychotic-like experiences (PLEs), 21.8% had clinically significant anxiety syndrome, 34.0% had clinically significant depressive syndrome, and 63.6% experienced at least one type of traumatic exposure during their childhood, with physical neglect being the most common. Compared to outpatients with HIV, prisoners living with HIV reported more severe mental health problems and a higher frequency of childhood trauma, with childhood trauma in turn predicting higher risk for mental health problems. Similarly, among outpatients living with HIV, both childhood emotional and sexual abuse had predictive effects on all the three mental health problems.

**Conclusions:**

The study suggests that PLWH have higher risk of anxiety, depression and PLEs, and childhood trauma could serve as predicting factors for such risks. In addition, childhood trauma may play distinct roles in predicting the risk for the mental health problems, depending on different subgroup of PLWH.

## Background

A great number of studies have showed that people living with HIV (PLWH) have experienced considerable degree of psychological distress. Almost 20% of PLWH suffer from an anxiety disorder at the clinical level [1], and 6.5 ~ 75% experienced mood or depressive disorders [2, 3]. Additionally, there is a high clinical prevalence of HIV-related psychosis, and the incidence of psychiatric syndrome in patients with HIV spectrum disorders ranges from 0.2 to 15%. The severity of psychiatric symptoms may increase with the progression of HIV disease [4]. It has been demonstrated that mental health status of PLWH not only significantly affects ART adherence and quality of life, but is also associated with negative experiences and behaviors, such as social isolation and stigma [5]. Notably, depressive symptoms have a particularly negative effect on adherence to ART, increasing HIV-related morbidity and mortality [6]. Meanwhile, together with stress, depressive symptoms are more likely associated with a decreased cluster of differentiation 4 (CD4) cell count and increased viral load [7, 8].

People in prisons and other closed settings are defined by WHO as a key population group who are at increased HIV risk in all countries and regions. It is reported that HIV prevalence of people in prisons is approximately five times higher than the general population [9]. In China, with the implementation of the “Four Frees and One Care***”*** policy [10], prisoners living with HIV are taken into centralized custody in a designated prison for standardized ART treatment. How to optimize the special disease management and how to improve the quality of life has become the focus of concern for government officers, especially prison administrators. However, related research on mental health problems among prisoners living with HIV is still limited in China.

Childhood trauma is one of the most critical risk factors for mental health problems [11, 12] and criminal behaviors [13]. Childhood trauma has been generally considered to be a destabilizing factor leading to the onset of psychotic symptoms and increasing biological vulnerability to the development of psychiatric disorders [14]. A growing number of researchers have identified that PLWH experience more childhood trauma than the general population [15]. Fear of violence and physical abuse has negative effects on the mental health of HIV-infected men who have sex with men (MSM) [16]. Moreover, Wang et al. have found that childhood trauma was a risk factor for recidivism among male prisoners [17]. However, the internal association between childhood trauma and mental health in prisoners living with HIV has not been fully clarified.

Psychotic-like experiences (PLEs) were also included in our study as measurable variables, with PLEs generally defined as positive symptoms of psychosis at the subclinical level [18]. PLEs have been demonstrated to be a predictive risk factor for later development of psychotic disorders [19, 20], as well as non-psychotic disorders [21]. However, PLEs among the population of PLWH, especially prisoners living with HIV, has barely been studied. In this study, we hypothesized that there would be higher rates of PLEs among the PLWH, especially among prisoners living with HIV.

To further explore these connections, we carried out a cross-sectional survey to determine prevalence of anxiety, depression and PLEs, as well as their associations with childhood trauma, among the PLWH in China. Meanwhile, we attempted to identify specific mental health characteristics among the two major subgroups of PLWH in our survey: prisoners living with HIV and outpatients living with HIV.

## Materials and method

### Participants

The sample of PLWH was recruited from Hunan Province, China, including outpatients and prisoners. Outpatients living with HIV were served by the HIV/AIDS clinic of the First Hospital of Changsha, which acts as the government appointed HIV-treatment Hospital for Hunan province. Prisoners living with HIV were recruited from two designated prison facilities for prisoners living with HIV (one was a women’s prison; another was a men’s prison) in Hunan.

All participants (or their guardians, if age < 18) signed the informed consent. The participants who were unable to understand the questionnaires or had a history of psychiatric disorders prior to their HIV diagnosis were excluded from the study.

### Procedure

Outpatients living with HIV were recruited from the patient waiting room at the HIV/AIDS clinic of the First Hospital of Changsha from March 2019 to June 2019. The outpatients completed the questionnaire by themselves and/or with the help of one research assistant. Prisoners living with HIV were recruited in a meeting room of the two chosen prisons from August 2019 to September 2019. One researcher and two prison guards accompanied them during the whole process, helping answer procedural questions. The survey was under the principle of voluntary participation, and informed consent was obtained from all participants before starting the survey. Participants could withdraw from the study at any time if they felt discomfort. We offered remuneration of 50 RMB to participants from the clinic. For the incarcerated participants, we did not offer remuneration, but we offered free group psychotherapy services.

The survey was conducted anonymously, and all data was kept entirely confidential. The investigation was carried out in accordance with the latest version of the Declaration of Helsinki and approved by the Ethics Committees of Xiangya Nursing School of Central South University (No.2018007). Under the original agreement, the names of the prisons and information about the prisoners’ crime were not reported here.

### Measures

#### PLEs

The 8-item Positive Subscale of the Community Assessment of Psychic Experiences (CAPE-P8) [22, 23] was used to assess the PLE frequency in the past month. CAPE-P8 originated from CAPE-42 [24], which addresses the following domains: delusional experiences (6 items) and hallucinatory experiences (2 items). In terms of frequency, responses to items range from 0-never, 1-sometimes, 2-often, to 3-nearly always. The higher scores indicate an increased level of frequency of PLEs. The Chinese version of CAPE-P8 had adequate psychometric properties [25]. The Cronbach’s alpha was 0.86 in the current sample.

### Anxiety

The 7-item Generalized Anxiety Disorder scale (GAD-7) [26] scale was used to measure levels of anxiety in the past two weeks. Responses to items range from 0 (not at all) to 3 (nearly every day). Higher the total scores indicate higher the levels of anxiety. The cut-off value of 10 represents identified cases of clinically anxiety, with acceptable sensitivity (89%) and specificity (82%). In this study, the internal consistency of the scale was high, with a Cronbach’s alpha of 0.94.

### Depression

The 9-item Patient Health Questionnaire (PHQ-9) was used to assess depressive symptoms within the past two weeks [27]. Responses to items range from 0-not at all, 1- several days, 2- more than half the days, to 3- nearly every day, with higher total score showing more severe depressive symptoms. Total score ≥ 10 had a sensitivity of 88% and a specificity of 88% for clinical level of depression. The Cronbach’s alpha in this sample was 0.92.

### Childhood trauma

The 28-item Childhood Trauma Questionnaire (CTQ) was used to measure self-reported experiences of childhood trauma before 16 years of age [28]. The CTQ describes five domains: emotional abuse (EA), physical abuse (PA), sexual abuse (SA), emotional neglect (EN) and physical neglect (PN). Higher CTQ scores indicate a higher level of childhood trauma experienced. Based on cut-off values of severe trauma exposure (EA ≥ 13, PA ≥ 10, SA ≥ 8, EN ≥ 15, PN ≥ 10), the existence of severe childhood trauma history can be identified [28]. In this sample, Cronbach’s alpha for the total score was 0.77.

### Statistical analysis

Data analyses were conducted using IBM SPSS Statistics version 22.0. Descriptive statistics were performed for socio-demography, HIV-related characteristics, childhood trauma and mental health characteristics. Any CAPE-P8 item with response of 2 (often) or 3 (nearly always) was counted and referenced as frequently suffers from PLEs [29]. A cut-off score of 10 on the GAD-7 and PHQ-9 was used to respectively split the anxiety and depression groups [26, 27]. Due to the limited sample size, the data in this study did not show the expected normal distribution (*p* < 0.05). Thus, correlation analysis was conducted with Spearman’s correlation coefficient to investigate associations among GAD-7, PHQ-9, CAPE-P8 and CTQ in the PLWH. The χ^2^ test and Independent sample t test were conducted to explore the differences in socio-demography, HIV-related characteristics, and childhood trauma of different PLWH subgroups. A series of univariate logistic regression analyses were performed to determine the associations between each independent variable (socio-demography and HIV-related characteristics) in Table 1 and mental health problems. Binary logistic regression was used to assess the associations between mental health problems (PLEs, anxiety, and depression) and childhood trauma, controlling for other socio-demographics and HIV-related characteristics. The odds ratios (OR) and 95% confidence intervals (95% CI) were calculated. *P*-value of less than 0.05 was considered to be statistically significant.

**Table 1:** Descriptive statistics of socio-demographic and HIV-related characteristics

	Overall *n* = 533	Prisoners n = 213	Outpatients n = 320	χ^2^/t	P
Age (years), M(SD)	33.01(9.93)	40.52(8.96)	28.12(7.06)	16.64	<.001
Education (years), M(SD)	12.41(3.82)	9.02(2.75)	14.65(2.58)	−23.38	<.001
Duration of HIV Infection (months), M(SD)	62.31(49.68)	94.33(54.03)	39.74(30.38)	13.02	<.001
Male, n(%)	484(90.8)	179(84.0)	305(95.3)	19.47	<.001
Ethnic Han, n(%)	489(91.7)	189(88.7)	300(93.8)	4.25	.053
Residence location in rural, n(%)	203(38.1)	89(41.8)	94(29.4)	18.69	<.001
Unmarried, n(%)	352(66.0)	83(39.0)	269(84.1)	127.66	<.001
Route of infection, n(%)				111.24	<.001
Sexuality	287(53.8)	80(37.6)	207(64.7)		
Blood transfusion	8(1.5)	5(2.3)	3(0.9)		
Needle sharing/ take drugs	76(14.3)	71(33.3)	5(1.6)		
Unknown	162(30.4)	57(26.8)	105(32.8)		
Frequent PLEs	117(22.0)	59(27.7)	58(18.1)	6.84	.010
Depression	181(34.0)	94(44.1)	87(27.2)	0.12	.784
Anxiety	116(21.8)	48(22.5)	68(21.3)	16.37	<.001

Note: Frequent PLEs was referred by any item in CAPE-P8 with response with 2 (often) or 3 (nearly always)

Depression was referred by PHQ-9 with a cut-off value of 10

Anxiety was referred by GAD-7 with a cut-off value of 10

## Results

### Description of the sample

Five hundred fifty seven PLWH participated in the study. Participants with > 25% missing data were excluded (*n* = 24), leaving a total sample of 533, of which 40.0% (*n* = 213) were prisoners and 60% (*n* = 320) were outpatients. 90.8% (*n* = 484) were male, and participants aged from 16.0 to 68.0 years, with a mean age of 33.01 (SD = 9.93) years. Sexual behavior (53.8%) was the most common route of HIV infection in our sample, although there were still 1/3 of prisoners who suffered from HIV by sharing needles while injecting drugs. Notably, 30.4% (*n* = 164) participants reported that they didn’t know how to be infected by HIV. The average reported duration of HIV infection was 62.31 (SD = 49.68) months. 97.7% (*n* = 521) participants had received ART treatment, except for only 12 outpatients living with HIV. The differences of detailed socio-demographic and HIV-related characteristics between the prisoner and outpatient group were shown in Table 1.

### Mental health characteristics

Table 1 also showed 22.0% of respondents (*n* = 117) reported the presence of PLEs often or nearly-always. 34.0% (*n* = 181) had depression, and 21.8% (*n* = 116) participants were identified as anxiety. Compared with outpatients, prisoners living with HIV exhibited more frequent PLEs (27.7% vs. 18.1, χ^2^ = 6.84, *p* = .010). No significant difference in anxiety was identified between the prisoners and outpatients (22.5% vs. 21.3%, χ^2^ = .12, *p* = .784). In addition, prisoners living with HIV reported a higher proportion of depression than outpatients living with HIV (44.1% vs. 27.2%, χ^2^ = 16.37, *p* < .001). As shown in Table 2, lower education level was significantly related to poor mental health of PLWH, as well as residence location in rural as a risk factor for frequent PLEs.

**Table 2:** Univariate logistic regression of associations between mental health problems, socio-demographic and HIV-related characteristics [Crude OR (95% CI)]

	Frequent PLEs n = 117	Anxiety n = 116	Depression n = 181
Age (years)	1.02(0.99,1.04)	1.02(0.99,1.04)	1.03(1.01,1.05)^**^
Education (years)	0.87(0.82,0.92)^***^	0.95(0.89,0.99)^*^	0.91(0.87,0.96) ^***^
Duration of HIV Infection (months)	1.00(1.00,1.01)	1.00(1.00,1.01)	1.01(1.00,1.01)
Male, (Female as Ref.)	2.13(0.89,5.14)	2.11(0.87,5.08)	1.47(0.76,2.85)
Ethnic Han, (Others as Ref.)	1.53(0.67,3.54)	1.84(0.76,4.46)	1.60(0.79,3.24)
Residence location in rural, (Urban as Ref.)	1.76(1.12,2.75)^*^	1.41(0.91,2.19)	1.33(0.92,1.94)
Unmarried, (Married as Ref.)	0.82(0.83,1.25)	1.13(0.73,1.75)	0.91(0.62,1.33)
Route of infection, (Sexuality as Ref.)			
Blood transfusion	1.29(0.25,6.55)	1.29(0.25,6.55)	1.31(0.31,5.62)
Needle sharing/ take drugs	1.48(0.83,2.63)	1.48(0.83,2.63)	1.68(1.00,2.82)
Unknown	1.07(0.67,1.71)	1.03(0.64,1.65)	1.13(0.75,1.70)

Note: OR: Odd-Ratio; CI: Confidence interval; ^*^p < .05; ^**^p < .01; ^***^p < .001

### Childhood trauma

Three hundred and thirty-nine (63.6%) participants experienced at least one type of traumatic exposure during their childhood, and the most common childhood trauma was PN (49.9%). Compared to outpatients living with HIV, prisoners living with HIV had higher proportions of exposure to childhood trauma, except for sexual abuse (*p* > .05) (see Table 3).

**Table 3:** Childhood trauma in PLWHs [n(%)]

	Overall n = 533	Prisoners n = 213	Outpatients n = 320	χ^2^	p
EA	32(6.0)	21(9.9)	11(3.4)	9.35	0.003
PA	58(10.9)	41(19.2)	17(5.3)	25.61	< 0.001
SA	137(25.7)	57(26.8)	80(25.0)	0.21	0.69
EN	192(36.0)	103(48.4)	89(27.8)	23.42	< 0.001
PN	266(49.9)	145(68.1)	121(37.8)	46.85	< 0.001
Any type of trauma	339(63.6)	160 (75.1)	179(55.9)	20.32	< 0.001

Note: *EA* emotional abuse, *PA* physical abuse, *SA* sexual abuse, *EN* emotional neglect, *PN* physical neglect

### Correlation analysis

CTQ total score showed significant positive correlations with the total scores of CAPE-P8 (r = 0.34, *p* < .001), GAD-7 (r = 0.25, *p* < 0.001), and PHQ-9 (r = 0.31, *p* < .001).

### Predicting effects of childhood trauma on mental health characteristics

Odds ratios (OR) and confidence intervals (CI) for each potential predicting variable were listed in Table 4. Three types of traumatic exposure were identified as significant predictors among prisoners living with HIV, including PN on PLEs (OR = 3.19, CI = 1.09–9.35), EA on anxiety (OR = 7.77, CI = 1.78–33.84), SA on anxiety (OR = 2.50, CI = 1.01–6.23) and depression (OR = 2.22, CI = 1.02–4.87). For outpatients living with HIV, EA and SA showed significantly positive effects on PLEs (EA: OR = 5.94, CI = 1.37–25.72; SA: OR = 2.82, CI = 1.31–6.09), anxiety (EA: OR = 5.36, CI = 1.31–21.84; SA: OR = 2.96, CI = 1.46–6.00), and depression (EA: OR = 8.64, CI = 1.83–40.79; SA: OR = 2.50, CI = 1.29–4.82), while PA, EN, and PN lacked significant predictive effects on the mental health of this sample.

**Table 4:** Results of the logistic regression in PLWHs^a^

		Frequent PLEs		Anxiety			Depression
		OR	CI (95%)	OR	CI (95%)	OR	CI (95%)
Prisoners	EA	2.10(.277)	0.55,8.04	**7.77(.006)**	1.78,33.84	4.01(.070)	0.89,18.06
	PA	1.08(.881)	0.38,3.08	1.46(.511)	0.47,4.51	1.49(.422)	0.56,3.98
	SA	1.97(.107)	0.86,4.50	**2.50(.048)**	1.01,6.23	**2.22(.046)**	1.02,4.87
	EN	1.52(.319)	0.67,3.47	0.64(.370)	0.25,1.68	0.76(.459)	0.36,1.59
	PN	**3.19(.034)**	1.09,9.35	3.47(.052)	0.99,12.16	1.24(.590)	0.57,2.72
Outpatients	EA	**5.94(.017)**	1.37,25.72	**5.36(.019)**	1.31,21.84	**8.64(.006)**	1.83,40.79
	PA	0.83(.803)	0.19,3.57	1.41(.616)	0.37,5.33	0.36(.157)	0.08,1.49
	SA	**2.82(.008)**	1.31,6.09	**2.96(.003)**	1.46,6.00	**2.50(.006)**	1.29,4.82
	EN	2.24(.067)	0.94,5.32	1.67(.231)	0.72,3.89	1.84(.120)	0.85,3.96
	PN	0.70(.410)	0.30,1.63	0.57(.189)	0.25,1.32	0.88(.727)	0.42,1.84

Note: ^a^adjusting for other socio-demographics and HIV-related characteristics

*EA* emotional abuse, *PA* physical abuse, *SA* sexual abuse, *EN* emotional neglect, *PN* physical neglect, *OR* Odd-Ratio, *CI* Confidence interval; Bold type indicates a significant odds ratio

## Discussion

Our study examined mental health characteristics (anxiety, depression and PLEs) and their associations with childhood trauma among two major subgroups of PLWH (prisoners and outpatients). It is the first pilot study to explore PLEs among PLWH, as well as the differences in mental health characteristics and factors of childhood trauma between the two subgroups of PLWH.

In our study, PLWH reported a high proportion of mental health problems, with 21.8% identified as high risk for anxiety and 34.0% identified as high risk for depression. This finding is consistent with previous studies. Based on the PHQ-9 and GAD-7 with a cut-off score of 10, data from across PLWH with ART treatment in U.S. also indicated elevated rates of depression (30%) [30]. In study of 667 PLWH (92% male) in China, 34.5% presented with moderate or severe depression, 27.3% presented with moderate or severe anxiety [31].

In addition, 22.0% PLWH experienced frequent PLEs in the current sample. Although PLEs are regarded as an important endophenotype in psychopathology, there is still lack of concern of their presence in PLWH. Notably, prisoners living with HIV showed a higher risk for PLEs and depression compared to outpatients in the study. These findings suggest more robust psychosocial interventions should be offered to this subgroup of PLWH because of their long-term incarceration and lack of family and societal support.

Adverse childhood experiences have been demonstrated to be common among populations of PLWH [15]. In our study, 63.6% PLWH experienced childhood trauma in the study, with physical neglect as the most common type (49.9%). Childhood trauma has been recognized as a significant predictor of HIV infection risk [32]. A cross-sectional study in South Africa showed those with childhood trauma were more likely to be at risk of HIV infection [33], which has been further demonstrated in another longitudinal study in rural South African youth [34]. One possible reason for this phenomenon is that people with childhood trauma are more likely to participate in a range of sexually risky behaviors [35], including unprotected sex and increased number of sex partners [36]. Another possible reason is that childhood trauma is closely related to drug use [37]. In our study, prisoners living with HIV reported more childhood trauma than outpatients. Previous studies also purported that adverse childhood experiences are critical risk factors for criminal behavior [38], with physical and sexual abuse increasing juvenile delinquency and adult criminality [39]. As previous studies have reported [40], we also found more childhood PN in prisoners living with HIV than outpatients. Lower levels of education and unhealthy lifestyles (e.g. drug abuse) may partly contribute to a higher likelihood of suffering from PN [41].

In line with previous studies [22], childhood trauma was found to be significantly and positively associated with PLEs, depression and anxiety in PLWH in this study. A strong body of evidence suggests that childhood trauma not only has a positive relationship with a broad range of mental health problems in the general population [42], but also exerts a significant and long-lasting impact on psychiatric disorders, worse quality of life and poor medication adherence, faster disease progression, and greater mortality rates among PLWH [43].

Furthermore, we explored the effects of five types of childhood trauma (EA, PA, SA, EN, PN) on mental health in different subgroup of PLWH, controlling for other socio-demographic and HIV-related characteristics. Our findings suggest that both EA and SA are risk factors predictive of PLEs, anxiety, and depression in outpatients living with HIV, as well as for anxiety in prisoners living with HIV. Prior studies have indicated that both EA and SA exhibit a stronger relationship with PLEs rather than other types of childhood trauma [44]. It has been suggested that childhood EA may decrease the ability of maladaptive cognitive emotion regulation, influencing current depression or anxiety severity [45]. Also, one study found that SA was closely associated with a lifetime diagnosis of anxiety disorder and major depression [46]. Notably, PN (i.e. the most common form of childhood trauma) was found to have a predictive effect only on PLEs in prisoners living with HIV, while there was no such effect on outpatients in our study. We speculate that childhood PN implies an adverse childhood environment (e.g. not have enough to eat), which interacts with genetic factors, resulting in a persistence of PLEs, and ultimately developing into poor outcomes [19].

Our findings indicated that childhood trauma plays an important role in PLWH’s mental health. PLWH who have experienced trauma or suffered from mental disorders face unique challenges in their HIV care engagement and ARV adherence [5, 47]. Starting from trauma or mental health problems may be an effective way to protect the health of HIV patients clinically. For example, improving AIDS Care after Trauma (ImpACT) is effective in reducing Post-traumatic stress disorder (PTSD) symptoms and increasing ART adherence motivation among PLWH [48]. Meanwhile, cognitive-behavioural therapy for adherence and depression (CBT-AD) has the potential to improve clinical depression, ART adherence and viral load for virally unsuppressed PLWH [49].

Some limitations should be considered for the study. There may be potential reporting bias, because detection of mental health problems relied on self-report questionnaires rather than clinical interviews in this study. In the future, clinical diagnosis should be involved to determine the prevalence rate of mental disorders. Meanwhile, the cut off score of 10 for GAD-7 and PHQ-9 are widely recognized among the general population. However, when it comes to the population of PLWH, such criterion should be treated with caution. For example, it has been demonstrated that PHQ-9 has high specificity but apparently low sensitivity for detecting MDD in HIV patients in low-income countries [50]. Thus, it remains necessary for further research to determine the performance of proven screening tools in the population of PLWH. Moreover, as for the prisoners: in terms of their management, all of the HIV-positive patients are imprisoned together, regardless of which law they may have broken, and thus we have no way of knowing when exactly they were infected. At the same time, for the clinic-based participants, it is difficult to know the timing of infection for a large majority of patients, unless it is due to certain circumstances (such as donating blood). Therefore, nature of prison sentences or length of stay and timing of infection this factor is difficult to accurately collect in our study. In addition, some other factors also may cause bias in our results, including significant differences in socio-demographic and HIV-related factors between the two subgroups, missing data, and impact of the life event of imprisonment for prisoners.

## Conclusion

In summary, this is the first exploratory study evaluating PLEs, depression and anxiety, as well as the predictive effects of childhood trauma on these mental health problems among the subgroups of PLWH in China. Importantly, our findings suggest that there are more severe mental health problems (PLEs and depression) and childhood trauma among prisoners living with HIV. Childhood emotional abuse and sexual abuse are strong predictive factors for PLEs, anxiety, and depression in PLWH. In particular, childhood physical neglect is a specific risk factor for PLEs among prisoners living with HIV. These findings support the importance of early intervention for childhood trauma, which may decrease occurring rates of PLEs, depression and anxiety, and even HIV infection.

## Data Availability

The datasets used and/or analyzed during the current study are available from the corresponding author (Xuan Ouyang: ouyangxuan@csu.edu.cn) on reasonable request.
